# Factors affecting hospital admission, hospital length of stay and new discharge destination post proximal humeral fracture: a retrospective audit

**DOI:** 10.1186/s12877-024-04928-z

**Published:** 2024-04-12

**Authors:** B. R. McDonald, S. Vogrin, C. M. Said

**Affiliations:** 1 Department of Physiotherapy, Western Health, St Albans, VIC Australia; 2 The University of Melbourne, Parkville, VIC Australia; 3 Australian Institute for Musculoskeletal Science, St Alban, VIC Australia; 4 Department of Medicine, Western Health, St Albans, VIC Australia

**Keywords:** Hospitalization, Length of stay, Discharge, Proximal humeral fracture

## Abstract

**Background:**

Outcomes following proximal humeral fracture (PHF) may be impacted by a range of clinical, fracture and premorbid factors. The aim of this study was to examine factors impacting hospital admission; length of stay (LOS) and new discharge destination for patients presenting to hospital with PHF.

**Methods:**

Retrospective audit conducted at a tertiary health service. Data was collected from adult patients presenting to hospital with a PHF over a 54-month period. Fractures that were pathological or sustained during admission were excluded. Univariable and multivariable logistic and negative binomial regression were used to explore factors associated with hospital admission, LOS and new discharge destination.

**Results:**

Data were analyzed from 701 participants (age 70 years (IQR 60, 81); 72.8% female); 276 (39.4%) participants required a hospital admission. New discharge destination was required for 109 (15.5%) participants, of whom 49 (45%) changed from home alone to home with family/friend(s). Greater comorbidities, as indicated by the Charlson Comorbidity Index score, were associated with hospital admission, longer LOS and new discharge destination. Premorbid living situations of home with family/friend(s) or from an external care facility were associated with a decreased likelihood of hospital admission, shorter LOS and reduced risk of a new discharge destination. Surgical treatment was associated with shorter LOS. Older age and dementia diagnosis were associated with a new discharge destination.

**Conclusions:**

Many factors potentially impact on the likelihood or risk of hospitalization, LOS and new discharge destination post PHF. Patients with greater comorbidities are more likely to have negative outcomes, while patients who had premorbid living situations of home with family/friend(s) or from an external care facility are more likely to have positive outcomes. Early identification of factors that may impact patient outcomes may assist timely decision making in hospital settings. Further research should focus on developing tools to predict hospital outcomes in the PHF population.

**Supplementary Information:**

The online version contains supplementary material available at 10.1186/s12877-024-04928-z.

## Background

Proximal humeral fracture (PHF) is a common fracture in people aged > 60 years old [1, 2], with PHF accounting for about 9.5% of fall-related fractures [2]. High prevalence of osteoporosis worldwide due to an aging population [3] has contributed to an increased incidence of PHF particularly in women [2]. Incidence of hospitalization due to PHF in Australia increased from 26.8 per 100,000 person-years in 2008 to 45.7 per 100,000 person-years in 2017 [1]. Treatment associated with PHF is also costly, with median hospitalization costs being US$16,447 for surgically managed patients and US$7226 for conservatively managed patients [4]. Hospital admission and hospital length of stay (LOS) are two outcomes that quantify the amount of care a patient needs post PHF. Currently little is known about what factors are associated with hospital admission post PHF [5, 6]. Hospital LOS has been used as a secondary outcome to evaluate outcomes following different surgical treatment methods [4, 7–12], however the impact of other factors on LOS post PHF has been less explored. While increased LOS can be indicative of the complexity of a patient [13], it may also be reflective of “inefficient hospital processes” ([14] p12); treatment delays and poor discharge planning [14]. Early identification of people at risk of longer LOS may assist with timely discharge planning. Another way outcomes can be evaluated is whether a person can return to their premorbid living situation following hospital discharge. Previous evidence suggests that factors such as older age [15–19]; gender [16, 18]; comorbidities [15–18]; premorbid function [18, 19]; LOS [15, 16, 19]; concomitant fractures [15]; acute medical complications [15, 16] and/or surgical treatment [15, 19, 20] may predict discharge destination, however these studies did not include premorbid living situation (i.e. the premorbid presence or lack of family or friends) as a factor. Understanding what factors may affect patient discharge destination post PHF may improve discharge planning following hospitalization, ultimately improving patient care and reducing health care costs.

There are several factors that may impact patient hospitalization, LOS and discharge destination post PHF that have previously not been explored. Orthopedic restrictions (weight-bearing restrictions and/or use of sling) following both surgical and conservative treatments can prevent the use of gait aids and thus impact on a patient’s mobility status. Similarly, a patient’s upper-limb dominance or premorbid ability to manage their personal self-care may further limit their functional independence. In addition, while LOS progressively increases with subsequent admissions due to osteoporotic fracture [21], previous literature has not analyzed how osteoporosis influences hospital outcomes specifically post PHF. Therefore, the aims of this research are to examine in patients with PHF factors associated with (1) hospital admission; (2) LOS for patients admitted to hospital and (3) new discharge destination for patients that both present and/or are admitted to hospital.

## Methods

### Study design and setting

This retrospective audit was conducted at Western Health, a single large tertiary health service in Australia with three primary hospital campus locations (Footscray Hospital, Sunshine Hospital and Williamstown Hospital). The community within the Western Health catchment is multicultural and socioeconomically diverse, with more than 40% born outside of Australia [22]. In addition, people within this community have relatively higher burden of chronic diseases, such as diabetes and stroke, compared to the rest of Australia [23]. Data were extracted from a 54-month period between January 2014 and July 2018.

### Participants

**Fig. 1:**
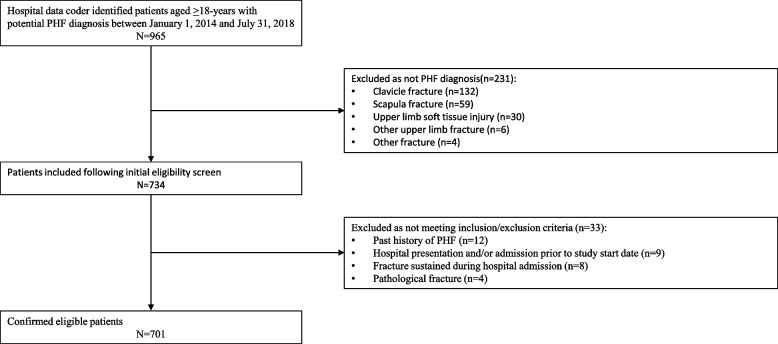
CONSORT diagram

The cohort of interest was adults presenting to hospital with a diagnosis of PHF. Inclusion criteria included aged 18-years or over, with a principal diagnosis of PHF at hospital presentation (defined as an admission to an emergency department (ED)) or admission (defined as a minimum of a one-night stay in hospital). Exclusion criteria included fractures that were classified as pathological or sustained during hospital admission. The hospital admission data base was searched to identify all patients treated at hospital with diagnostic related group (DRG) codes which may have indicated PHF during the time period (S42.20 fracture of upper end of humerus, part unspecified; S42.21 fracture of head of humerus; S42.22 fracture of surgical neck of humerus; S42.23 fracture of anatomical neck of humerus; S42.24 fracture of greater tuberosity of humerus; S42.29 fracture of other and multiple parts of upper end of humerus). This search identified 965 patients. The medical records of these patients were audited for eligibility, of which 701 were confirmed eligible. Reasons for exclusion are outlined in Fig. 1.

### Data collection

The researcher auditor extracted data from medical records and imaging reports utilizing a specifically designed data audit tool (see Additional file 1). Data collected included:

1. Primary outcome measures:
  Hospital admission: defined as a minimum of a one-night stay in hospital, including acute inpatient and/or subacute (rehabilitation or transitional care) ward(s).
  LOS (assessed in days for patients admitted to hospital and calculated by subtracting the admission date from the discharge date).
  Discharge destination: defined as either home alone; home with family/friend(s) (who resided with the patient); external care facility (residential aged care or supported accommodation); external health-care service or death.
2. Secondary factors, which may impact these outcomes were also assessed and classified in three domains: clinical, fracture and premorbid status factors. Further details are provided in Table 1.

**Table 1:** Secondary factors

Secondary factors		
**Clinical factors**	**Fracture factors**	**Premorbid status factors**
Age^a^	Fracture severity (displaced or non-displaced and open or closed)^a^	Premorbid living situation (home alone, home with family/friend(s) or external care facility (supported residential or residential aged care services))^a^
Gender^a^	Treatment method (conservative; surgical or conservative and surgical)^a^	Premorbid level of mobility (independent or assistance by another or others)^a^
Additional principal acute medical diagnoses^b^	Orthopedic restrictions (restrictions or minimal restrictions)^a,c^	Premorbid use of gait aid (nil aid or SPS/FAC; walking frame or standing machine/sling hoist)^a^
Additional significant injuries, such as bilateral PHFs, other fractures or brain hemorrhage^b^		Premorbid level of personal self-care (independent or assistance by another or others)^a^
Dementia diagnosis^b^		Dominance of upper limb affected (dominant, non-dominant or not documented))^a^
Osteoporosis diagnosis^b^		
Charlson Comorbidity Index (CCI) score [24], retrospectively scored^b^		

*Abbreviations*: *SPS* Single point stick, *FAC* Forearm crutch

^a^As documented in the imaging report or medical records

^b^As documented in the medical record discharge summary

^c^Restrictions were defined as immobilization in a sling/brace and/or non-weight-bearing orders; minimal restrictions defined as no/limited immobilization in a sling and/or an alternative weight-bearing status

### Statistical analysis

Descriptive statistics (medians with interquartile ranges or frequency and percentages) were calculated for baseline characteristic data. Factors associated with the three primary outcomes were explored using logistic regression for hospital admission, negative binomial regression for LOS and logistic regression for new discharge destination. Variables with *p* < 0.100 on univariable regression were included in the multivariable analysis. Results are expressed as odds ratios (OR) or incidence rate ratios (IRR) respectively with 95% confidence intervals (CI). There was no missing data for continuous variables. Missing categorical data were classified as a separate category (not documented); recorded using the highest level of function (independent for premorbid level of mobility; nil aid for use of gait aid; independent for premorbid level of personal self-care) or assumed no change in discharge destination compared to the premorbid living situation. This approach was taken as usual clinical practice in the busy environments, such as the ED, was to document only essential information necessary for patient management and discharge planning. Therefore, the absence of documentation on mobility, function or discharge destination indicated no deficits or changes in these areas. All analyses were conducted using Stata 16.1 software (StataCorp. 2019. College Station, TX).

## Results

A total of 701 participants were included within the study. The study cohort had a median age of 70 years (IQR 60, 81) with 614 (87.6%) participants over 50 years of age and 510 (72.8%) participants were female. Further participant baseline characteristics are summarised in Table 2.

**Table 2:** Baseline characteristics of all participants

	**All participants (** ***n*** ** = 701)**
**Clinical factors**	
Age, y median (IQR)	70 (60, 81)
Gender (female)	510 (72.8%)
Additional principal acute medical diagnosis	112 (16.0%)
Additional significant injuries	77 (11.0%)
Dementia diagnosis	86 (12.3%)
Osteoporosis diagnosis	156 (22.3%)
CCI score, median (IQR)	1 (0, 2)
**Fracture factors**	
Fracture severity	
Displaced	551 (78.6%)
Closed	701 (100%)
Treatment method	
Conservative management only	581 (82.9%)
Surgical management only	50 (7.1%)
Conservative then surgical management	70 (10.0%)
Orthopedic restrictions	
Restrictions	654 (93.3%)
**Premorbid status factors**	
Living situation	
Home alone	203 (29.0%)
Home with family/friend(s)	453 (64.6%)
External care facility	45 (6.4%)
Level of mobility	
Independent	654 (93.3%)
Gait aid use	
Nil or SPS/FAC	571 (81.5%)
Walking frame	122 (17.4%)
Standing machine/sling hoist	8 (1.1%)
Level of personal self-care	
Independent	600 (85.6%)
Upper-limb dominance	
Dominant	246 (35.1%)
Non-dominant	229 (32.7%)
Not documented	226 (32.2%)

*Abbreviations*: *y* Year, *IQR* Interquartile range, *CCI* Charlson Comorbidity Index, *SPS* Single point stick, *FAC* Forearm crutch

### Factors associated with hospital admission

Four hundred and twenty-five (60.6%) participants required only a hospital presentation, while the remaining 276 (39.4%) required a hospital admission. Table 3 presents the results of the univariable and multivariable analysis. After adjusting for confounders, factors associated with increased likelihood of hospital admission included additional principal acute medical diagnosis (OR 32.46); additional significant injuries (OR 5.59); osteoporosis diagnosis (OR 4.99); higher Charlson Comorbidity Index (CCI) score (OR 1.52); displaced fracture (OR 2.31) and premorbid use of a walking frame (OR 2.86). A decreased likelihood of hospital admission (i.e., more likely treated in ED, not admitted to hospital) were associated with the premorbid living situations of home with family/friend(s) (OR 0.38) or external care facility (OR 0.01).

**Table 3:** Association Between Factors and Hospital Admission (as Expressed as Odds Ratio (OR) with 95% Confidence Intervals (CI) and Probability Significance Value (p)); Association Between Factors Total LOS^1^ (as Expressed as Incidence Rate Ratio (IRR) with 95% CI and *p* value) and New Discharge Destination^2^ (as Expressed as OR with 95% CI) and *p* value)

Odds of hospital admission			Total LOS^1^ (*n* = 701)		New discharge destination^2^ (*n* = 701)	
	**Univariable analysis, OR (95% CI),*p*value**	**Multivariable analysis, OR (95% CI),*p*value**	**Univariable analysis, IRR (95% CI),*p*value**	**Multivariable analysis, IRR (95% CI),*p*value**	**Univariable analysis, OR (95% CI),*p*value**	**Multivariable analysis, OR (95% CI),*p*value**
**Clinical factors**						
Age, y median (IQR)	1.05 (1.03, 1.06), < **0.001**	1.02 (1.00, 1.04), 0.051	1.02 (1.01, 1.03), < **0.001**	1.00 (0.99, 1.01), 0.387	1.10 (1.07, 1.12), < **0.001**	1.09 (1.06, 1.12), < **0.001**
Gender (male)	1.02 (0.73, 1.44), 0.89		0.85 (0.65, 1.12), 0.253		0.72 (0.44, 1.17), 0.184	
Additional acute principal medical diagnosis	36.67 (16.72, 80.43), < **0.001**	32.46 (12.53, 84.08), < **0.001**	1.74 (1.36, 2.22), < **0.001**	1.39 (1.12, 1.74), **0.003**	3.75 (2.36, 5.95), < **0.001**	1.34 (0.70, 2.56), 0.38
Additional significant injuries	9.23 (5.06, 16.82), < **0.001**	5.59 (2.45, 12.74), < **0.001**	1.60 (1.21, 2.12), **0.001**	1.61 (1.26, 2.06), < **0.001**	2.70 (1.59, 4.59), < **0.001**	1.52 (0.74, 3.11), 0.253
Dementia diagnosis	2.82 (1.77, 4.49), < **0.001**	1.25 (0.49, 3.17), 0.644	1.45 (1.07, 1.98), **0.017**	1.18 (0.88, 1.58), 0.258	2.97 (1.78, 4.96), < **0.001**	2.44 (1.13, 5.23), **0.022**
Osteoporosis diagnosis	9.55 (6.23, 14.64), < **0.001**	4.99 (2.71, 9.21), < **0.001**	1.71(1.35, 2.18), < **0.001**	1.38 (1.10, 1.74), **0.005**	3.20 (2.08, 4.93), < **0.001**	0.90 (0.49, 1.64), 0.725
CCI score, median (IQR)	1.68 (1.50, 1.87), < **0.001**	1.52 (1.28, 1.81), < **0.001**	1.16 (1.09, 1.23), < **0.001**	1.13 (1.06, 1.19), < **0.001**	1.32 (1.20, 1.46), < **0.001**	1.23 (1.06, 1.43), **0.005**
**Fracture factors**						
Fracture severity						
Non-displaced	Ref	Ref	Ref			
Displaced	2.15 (1.44, 3.21), < **0.001**	2.31 (1.12, 4.80), **0.024**	1.03 (0.72, 1.46), 0.88			
Treatment method						
Conservative management	Ref		Ref	Ref	Ref	Ref
Surgical management	N/A		0.37 (0.27, 0.51), < **0.001**	0.56 (0.42, 0.76), < **0.001**	0.95 (0.43, 2.09), 0.899	2.27 (0.78, 6.58), 0.133
Conservative then surgical management	0.62 (0.35, 1.09), 0.096	1.70 (0.80, 3.63), 0.168	0.42 (0.26, 0.68), < **0.001**	0.68 (0.44, 1.06), 0.092	0.30 (0.11, 0.85), **0.023**	0.97 (0.30. 3.16), 0.966
Orthopedic restrictions						
Minimal restrictions	Ref	Ref	Ref	Ref	Ref	
Restrictions	0.34 (0.18, 0.63), **0.001**	0.59 (0.18, 1.89), 0.376	0.39 (0.29, 0.53), < **0.001**	1.13 (0.80, 1.59), 0.495	1.06 (0.46, 2.42), 0.898	
**Premorbid status factors**						
Living situation						
Home alone	Ref	Ref	Ref	Ref	Ref	Ref
Home with family/friend(s)	0.54 (0.39, 0.76), < **0.001**	0.38 (0.22, 0.67), **0.001**	0.69 (0.54, 0.88), **0.003**	0.67 (0.53, 0.83), < **0.001**	0.13 (0.08, 0.20), < **0.001**	0.08 (0.05, 0.15), < **0.001**
External care facility	0.21 (0.09, 0.46), < **0.001**	0.01 (0.00, 0.06), < **0.001**	0.24 (0.11, 0.51), < **0.001**	0.20 (0.10, 0.40), < **0.001**	0.04 (0.01, 0.28), **0.001**	0.00 (0.00, 0.04), < **0.001**
Level of mobility						
Independent	Ref		Ref		Ref	
Assistance	1.26 (0.70, 2.29), 0.441		0.96 (0.61, 1.53), 0.872		1.12 (0.51, 2.47), 0.773	
Gait aid use						
Nil aid or SPS/FAC	Ref	Ref	Ref	Ref	Ref	Ref
Walking frame	6.01 (3.87, 9.34), < **0.001**	2.86 (1.24, 6.57), **0.013**	1.40 (1.08, 1.82), **0.011**	0.98 (0.74, 1.28), 0.855	2.90 (1.83, 4.60), < **0.001**	0.75 (0.37, 1.53), 0.433
Standing machine/sling hoist	2.14 (0.53, 8.64), 0.287	3.02 (0.29, 31.37), 0.355	0.93 (0.34, 2.60), 0.896	0.59 (0.24, 1.46), 0.251	0.99 (0.12, 8.16), 0.993	1.24 (0.09, 16.10), 0.871
Level of personal self-care						
Independent	Ref	Ref	Ref	Ref	Ref	Ref
Assistance	2.60 (1.69, 4.00), < **0.000**	1.96 (0.72, 5.34), 0.186	1.33 (0.99, 1.78), 0.060	1.39 (1.02, 1.89), **0.037**	2.02 (1.22, 3.36), **0.007**	2.13 (0.95, 4.80), 0.067
Upper-limb dominance						
Non-dominant	Ref		Ref		Ref	
Dominant	1.60 (1.11, 2.30), **0.012**		0.95 (0.73, 1.25), 0.723		1.23 (0.76, 1.99), 0.391	
Not documented	0.61 (0.41, 0.90), **0.013**		0.40 (0.29, 0.56), < **0.001**		0.73 (0.43, 1.24), 0.245	

Blank cell = results not analyzed. Bold indicates statistically significant *p* value

*Abbreviations*: *y* Years, *IQR* Interquartile range, *CCI* Score, Charlson Comorbidity Index, *N/A* Not applicable, *SPS* single point stick, *FAC* Forearm crutch

^1^Only participants who were admitted to hospital (*n* = 276) were included in the analysis for factors associated with an increased risk of longer total LOS

^2^All participants (*n* = 701) who presented to or were admitted to hospital were included in the analysis for factors associated with a new discharge destination

### Factors associated with total LOS for patients admitted to hospital

The median LOS for the 276 participants admitted to hospital was 23 days (IQR 7, 48.5). Table 3 presents the results of the univariable and multivariable analysis. Factors associated with an increased risk of a longer LOS included additional principal acute medical diagnosis (IRR 1.39); additional significant injuries (IRR 1.61); osteoporosis diagnosis (IRR 1.38); higher CCI score (IRR 1.13) and premorbid status of assistance with personal self-care (IRR 1.39). Factors that were associated with a decreased risk of longer LOS included surgical treatment (IRR 0.56) and the premorbid living situations of home with family/friend(s) (IRR 0.67) or an external care facility (IRR 0.20).

### Discharge destination outcomes

Five hundred and ninety-two (84.5%) participants were discharged to the same discharge destination as their premorbid living situation. Of the 109 (15.5%) participants who were discharged to a new discharge destination, 49 (45%) changed from home alone to home with family/friend(s) and 29 (26.6%) changed from home alone or with family/friend(s) to an external care facility. Further detail of the new discharge destination changes can be found within Additional file 2.

### Factors associated with new discharge destination for all patients

Statistical analysis of factors associated with discharge destination are displayed in Table 3. Factors associated with an increased risk of a new discharge destination included older age (OR 1.09); dementia diagnosis (OR 2.44) and higher CCI score (OR 1.23). Premorbid living situations of home with family/friend(s) or from an external care facility were found to be associated with a reduced risk of a new discharge destination (OR 0.08 and OR 0.00 respectively).

## Discussion

Discharge planning for patients presenting to hospital post PHF is a complex task. Clinicians need to consider what factors contribute to this decision making to provide patients with PHF the best care and reduce health care costs. We found that approximately one in three patients presenting with PHF required hospital admission, and one in seven were discharged to a different living situation. We also identified that patients with greater comorbidities (as indicated by the CCI) were more likely to require hospital admission, a longer LOS and new discharge destination. Conversely, compared with patients who lived home alone premorbidly, patients who were previously living at home with family/friend(s) or from an external care facility were less likely to require hospital admission, have a longer LOS or have a new discharge destination. In addition, several other clinical, fracture or premorbid factors were associated with one or more of the primary outcomes.

Our finding that a higher CCI score was associated with worse outcomes is in keeping with the limited existing literature, which found that an increased CCI score is associated with hospital admission [5]; a longer LOS [6] and new nursing home admission on discharge [17]. Although the CCI has been suggested to be a valid mortality predictor tool for patients with PHF [25, 26], there has been some criticism that condition weightings are outdated [27]. By comparison, the Elixhauser comorbidity measure has been found to be superior to the CCI at discriminating inpatient mortality with PHFs [28]. Nonetheless, these findings highlight the importance of considering a person’s comorbidities when planning likely outcomes and suggest that a comorbidity index should be considered for inclusion in any predictive model.

In contrast, patients who were from home with family/friend(s) or from an external care facility had a decreased likelihood of hospital admission, a shortened LOS and a reduced risk of a new discharge destination compared with patients who previously lived home alone. Of note, of the 109 (15.5%) participants requiring a new discharge destination, 49 (45%) were people who transitioned from home alone to home with family/friend(s) and only 29 (26.6%) were people discharged to an external care facility. Comparison to the literature is limited as previous studies lacked detail on how the patient’s premorbid living situation changed on discharge [15–20], with most only assessing discharge to an external care facility as an outcome [15–17, 20]. This supports previous research, which found patients with PHF have a significantly lower risk of residential care placement compared to other fragility fractures (using hip fractures as a reference) [29]. Our findings likely reflect the increased care needs, either temporarily or permanently, that patients experience post PHF and highlights the importance of considering premorbid living situation in planning.

Surgical treatment (compared to patients treated conservatively) was also found to be associated with a shorter LOS. Everyone who required surgery required hospitalization, with most patients discharged quickly post-operatively (median LOS was 5 days (IQR 3, 18), one in three were discharged in two nights or less). Conversely, not every patient who was treated conservatively required hospitalization. Conservatively managed patients admitted to hospital likely required admission due to other factors, such as comorbidities and additional medical diagnoses or injuries. These issues generally do not resolve quickly thus require a longer LOS, which may have included subacute admission. Our inclusion of these settings in our study’s LOS may also explain why our study contrasts to other recent literature, which found surgical treatment was associated with longer LOS [4, 12].

Factors associated with both hospital admission and longer LOS included osteoporosis diagnosis, additional principle acute medical diagnosis and additional significant injuries. Other studies [5, 6, 15, 17–19] have not explored the relationship between osteoporosis and outcomes; it may be an indicator of frailty which may explain the association with negative outcomes. The remaining results are in keeping with three previous studies, which found patients who had “polytrauma” ([5], p156) were more likely to be hospitalized and patients with additional acute medical issues post-operatively [30, 31] were associated with a longer LOS. These results conflict with one study conducted in a specialist trauma centre, which found no differences in LOS between patients with isolated PHFs and “concomitant fractures” ([32], p102). We believe patients with these additional medical diagnoses or significant injuries are more likely to need hospitalization and a longer LOS in order to receive further medical treatment and provide more time to recover and rehabilitate.

Additionally, we found some factors were only associated with either hospital admission or longer LOS. Our findings that displaced fracture and premorbid use of a frame were associated with hospitalization is supported by Myeroff et al. [6] who found Neer fracture classification (> 1 vs 1, i.e., displaced fracture) and premorbid use of a “cane/walker/wheelchair.” ([6], p4) for mobility were predictors of hospital admission. These patients presenting may be more likely to need hospitalization to ensure that the relevant medical specialities can decide the best treatment methods and/or allied health can provide input for management of mobility issues if gait aid use is restricted. We also found that patients who required assistance with premorbid personal self-care were associated with a longer LOS. While it is recognised that “most” ([33], p885) people require family or friends to assist with personal self-care following PHF injury [33], with previous literature only reviewing outcome measures to assess this function [34, 35], no research has investigated how premorbid personal self-care function influences hospital outcomes. People who needed assistance with personal self-care premorbidly are likely to have substantial care needs following PHF. These additional needs may necessitate a longer LOS to allow patients and/or carers to learn adaptive strategies and rehabilitate post fracture or obtain community based personal-care services.

Two factors, older age and dementia diagnosis, were found to be associated with new discharge destination. While direct comparison is difficult, this is in keeping with previous studies [15, 17–19, 36], which also found age and dementia diagnosis were associated with discharge to “short-term or long-term care facility” ([15], p1703), “new nursing home” ([17], p1604), or non-home discharge [18, 19, 36]. Although it appears that older age [15, 17, 19] and dementia [15] may be factors associated with discharge to a residential care facility post PHF, more research with clearly defined premorbid and discharge destination locations needs to explore this change directly. In addition, further research needs to explore the influence of dementia diagnosis on patient discharge destination outcomes post PHF, as this has only been analyzed in one previous study [15] which differed in its methods of diagnosis to our study. While conflicting results in a study by Wang, Youssef and Smerdely [17] found no association between cognitive impairment and a new nursing home admission post PHF [17] this may be explained by the severity of the condition. It also however raises the question on whether patients with PHF and a diagnosis dementia were deemed by clinicians to have limited capacity to make functional gains, therefore required a new discharge destination to ensure new caregivers at home or in an external care facility could take on their increased care needs. Further exploration of issues surrounding access to rehabilitation and discharge planning in people with dementia is warranted.

Improved understanding on factors that are associated with hospitalization, LOS and discharge destination post PHF will assist guiding development and implementation of a tool to assist decision making and discharge planning. Many of the factors we found to be associated with our primary outcomes may also be linked to patient frailty, therefore frailty indexes may be a more feasible way to help clinicians to accurately analyze patients. The current evidence however to support the use of frailty indexes is conflicting in patients post PHF. The modified Frailty Index (mFI-5) [37], which assesses similar comorbidities as the modified CCI (mCCI) in addition to functional status, has been found in patients post PHF surgery to be strongly associated with adverse events [38] or complications [39], while also predictive of readmission rates and the need for inpatient rehabilitation admission [39] and therefore risk of a longer LOS. In contrast, Yi et al. [38] found that both the mFI-5 and the mCCI had a limited ability to predict complications following PHF surgery. Another alternative tool includes the Score for Trauma Triage in the Geriatric and Middle-Aged (STTGMA) [40] which assesses patient age and CCI score, in addition to level of consciousness, the mechanism of injury and its severity at presentation [41]. The STTGMA tool may assist in “early decision making processes” ([40], p6) as one study found patients with PHF and high risk scores had a longer LOS, increased hospital needs and had fewer discharges directly home [40]. To date, the STTGMA has only been validated retrospectively [41] and the one study completed in patients with PHF only had a small sample size and was limited to adults 55-years or greater (as opposed to adults 18-years or greater) [40].

The main limitation of this study is that it was conducted retrospectively using data from a single health service. Results are likely reflective of people in a lower socioeconomic community. All data was collected based on documentation in the medical records and imaging reports utilising a specifically designed data audit tool. Missing or incorrect information may have caused errors in the data collected. Data were audited over a 54-month period. While there were no organizational changes that would have impacted on discharge planning over this time period, it is possible that local changes in clinical practice may have impacted LOS. Inspection of LOS data by year demonstrated no consistent changes in LOS. We were unable to include other confounding variables, such as ethnicity and pain, as these were not always recorded in a systematic way during the study time period. Nonetheless, the audit was conducted on a large sample of participants (*n* = 701) which has allowed the inclusion of multiple variables in the analysis. This will assist future researchers in the identification of factors to be included in the development of predictive tools.

## Conclusions

There are many factors that potentially impact on the likelihood or risk of hospital admission, LOS and new discharge destination post PHF. Patients with greater comorbidities had an increased likelihood of requiring hospitalization, a longer LOS and increased risk of a new discharge destination. In contrast, patients who had premorbid living situations of home with family/friend(s) or from an external care facility had decreased likelihood of hospitalization, a shortened LOS and a reduced risk of a new discharge destination. Once in hospital, patients who were treated surgically had a shorter LOS compared with those treated conservatively. Clinicians should consider early identification of factors that may impact hospital admission, LOS and a change in discharge destination to assist timely decision making and discharge planning in hospital settings. Our findings can be used to assist the development of tools to predict hospital outcomes in patients post PHF.

### Supplementary Information

**Supplementary Material 1.**

**Supplementary Material 2.**

## Data Availability

The data that support the findings of this study are available from Western Health but restrictions apply to the availability of these data, which were used under license for the current study, and so are not publicly available. Data are however available by contacting the corresponding author upon reasonable request and with permission of Western Health.
