# Supplementary material for: Factors affecting hospital admission, hospital length of stay and new discharge destination post proximal humeral fracture: a retrospective audit
Source: BMC Geriatr. 2024 Apr 12;24:334. doi: 10.1186/s12877-024-04928-z (PMC11015557; doi:10.1186/s12877-024-04928-z)
Supplement: Supplementary file 1 — Supplementary Material 1. [file 12877_2024_4928_MOESM1_ESM.pdf]

Case Record Form (CRF)

Patient Details

Study ID number:

DOB (date of birth):

Age:

Gender: ☐ **M** ☐ **F**

Discharge Destination and Length of Stay (LOS) Outcomes

**Admission date:**		
**Discharge date:**		
**ED admission**	☐Yes	☐No
**Acute ward admission**	☐Yes	☐No
**Subacute admission**	☐Yes	☐No
**Final discharge destination**	☐Home alone ☐Home with social supports (family/friends)<br>☐External Care Facility ☐Death during admission<br>☐ External Health Care Service	

Fracture and additional Injury Factors

**PHF Diagnosis**	☐Yes<br>☐No = Excluded from study (cease audit)
**Fracture severity**	☐Open ☐ Closed –non-displaced ☐ Closed –displaced<br>☐Not documented
**Pathological fracture**	☐Yes = Excluded from study (cease audit) ☐ No
**Location of fracture**	☐Whilst admitted in hospital = Excluded from study (cease audit)<br>☐Outside of Hospital
**Treatment method**	☐Surgical management ☐Conservative management
**Orthopaedic restrictions**	☐Restrictions ☐No/Minimal Restrictions

Initial documented orthopaedic management and orders

Any documented updates or changes to the orthopaedic management and orders

Additional significant injuries sustained (fractures; subdural haemorrhage)

☐No

☐Yes

Type(s):

Case Record Form (CRF)

Patient Premorbid Status Factors

Premorbid Living Situation ☐Home alone ☐External Care Facility
☐Home with social supports (family/friends)

Premorbid level of mobility ☐Independent ☐Supervision/Assistance/Dependent

Premorbid use of gait aid

☐Nil aid, single point stick (SPS) or forearm crutch (FAC)
☐Frame (two wheeled frame (2WF), four wheeled frame (4WW/4WF), gutter-frame (GF) or pick-up frame (PUF))
☐Non-ambulant

Premorbid level of personal self care ☐Independent ☐Supervision/Assistance/Dependent

Dominant Upper-limb affected ☐Yes ☐No ☐Not documented

Patient Clinical Factors

Additional principle medical diagnosis

☐No ☐Yes Diagnosis:________________________________

Charlson Comorbidity Index (CCI)

Score	Age/Condition
1	☐Age 50-59<br>☐Myocardial infarction (MI)<br>☐Congestive heart failure (CHF)<br>☐Peripheral vascular disease (PVD)<br>☐Cerebrovascular accident (CVA) or transient ischaemic attack (TIA).<br>☐Dementia<br>☐Chronic obstructive pulmonary disease (COPD)<br>☐Connective tissue disease<br>☐Peptic ulcer disease<br>☐Mild liver disease (without portal HTN, includes chronic hepatitis)<br>☐Diabetes without end-organ damage (excludes diet controlled alone)
2	☐Age 60-69<br>☐Hemiplegia<br>☐Moderate or severe renal disease<br>☐Diabetes with end-organ damage (retinopathy, neuropathy, nephropathy, or brittle diabetes)<br>☐Solid tumour without metastases<br>☐Leukaemia<br>☐Lymphoma
3	☐Age 70-79<br>☐Moderate or severe liver disease
4	☐Age $\geq$80
6	☐Metastatic solid tumour<br>☐AIDS (not just HIV positive).
**Total score**	

Diagnosis of dementia ☐Yes ☐No

Diagnosis of osteoporosis ☐Yes ☐No
