# Supplementary material for: Factors affecting hospital admission, hospital length of stay and new discharge destination post proximal humeral fracture: a retrospective audit
Source: BMC Geriatr. 2024 Apr 12;24:334. doi: 10.1186/s12877-024-04928-z (PMC11015557; doi:10.1186/s12877-024-04928-z)
Supplement: Supplementary file 2 — Supplementary Material 2. [file 12877_2024_4928_MOESM2_ESM.docx]

**Additional file 2 Table. New Discharge Destination (as Expressed as Count (Percentage)).**

| Discharge destination change | N (%) |
| --- | --- |
| From home alone to: | |
| - Home with family/friend(s) | 49 (45%) |
| - External care facility | 16 (14.7%) |
| - External health care | 9 (8.3%) |
| - Died | 2 (1.8%) |
| From home with family/friend(s) to: | |
| - Home alone | 2 (1.8%) |
| - External care facility | 13 (11.9%) |
| - External health care | 11 (10.1%) |
| - Died | 6 (5.5%) |
| From external care facility to: | |
| - Home with family/friend(s) | 1 (0.9%) |
